# The Bacteriomes of Ileal Mucosa and Cecal Content of Broiler Chickens and Turkeys as Revealed by Metagenomic Analysis

**DOI:** 10.1155/2016/4320412

**Published:** 2016-12-28

**Authors:** Shan Wei, Michael Lilburn, Zhongtang Yu

**Affiliations:** Department of Animal Sciences, The Ohio State University, Columbus, OH 43210, USA

## Abstract

The gastrointestinal (GI) bacteriome of poultry is important in host nutrition and health, but its diversity and composition remain poorly characterized. In this study we phylogenetically characterized the bacteriome in the cecal contents and ileal mucosa of chickens and turkeys using metagenomics empowered by pyrosequencing technique. >95% coverage of bacterial diversity was achieved except for the turkey ileal mucosa. Collectively, 3,401 and 125 operational taxonomy units (OTU, defined at a 0.03 phylogenetic distance) in chicken, and 1,687 and 16 OTUs in turkey were identified from the cecal content and the ileal mucosa, respectively. Besides those previously reported, 39 and 50 additional genera of bacteria were identified in the chicken and turkey cecal bacteriome, respectively. Although the GI bacteriomes of the same region in both species exhibited greater similarity than the bacteriomes of different regions within each species, broiler chickens and turkeys harbor a distinct intestinal bacteriome. Such difference may suggest different dietary interventions for bacteriome modulation for enhanced nutrient utilization and gut health. The results may also be useful in developing prebiotics, probiotics, and analytical tools (e.g., phylochips). We also determined the variation in the number of OTUs and variability between two independent pyrosequencing runs and two data processing pipelines.

## 1. Introduction

The poultry gastrointestinal (GI) tract harbors a dynamic microbial community consisting of a large number of species, primarily bacteria [1]. This bacterial community, or bacteriome, plays a pivotal role in the overall health and performance of poultry. The GI bacteria can be roughly classified as either pathogenic or commensal organisms [2]. Pathogenic bacteria can harm the host by causing localized or systemic infections and intestinal lesions [3] while commensal bacteria can benefit the host by providing nutrients, metabolic facilitation, and competitive exclusion [4, 5]. A better understanding of the bacterial composition and activity as well as the underlying mechanisms by which indigenous bacteria modulate the GI environment is needed to improve host health and feed utilization.

For many years, studies aimed at understanding the poultry GI bacteriome relied on classical cultivation techniques. During the past two decades, however, the 16S rRNA gene has been used as a primary biomarker for bacterial identification in various environments including the GI tract of poultry. This technique overcomes the limitation of culture-dependent methodologies thus potentially allowing for the identification of all the GI bacteria irrespective of their culturability. Studies using individual 16S rRNA gene clone libraries have provided valuable insight into the diverse GI bacteriome of poultry by producing high-quality sequences. However, these studies were limited to relatively small numbers of sequences that were affordable to researchers. Consequently, a comprehensive study looking into the diversity and composition of the GI bacteriome in poultry was not possible until next-generation sequencing (NGS) becomes available.

High-throughput NGS technologies have proven to be powerful tools for comprehensive analysis of complex bacteriomes [6, 7]. NGS technologies can generate large amounts of sequencing data at a relatively low cost. It also allows for sequencing of environmental DNA without a prior cloning step thereby eliminating cloning bias [8, 9]. The unprecedented sequencing capacity also allows for the identification of bacteria that are present in low abundance in a bacteriome. Although being replaced by other NGS technologies, the 454 pyrosequencing technology is the first NGS technology that has been widely used to analyze the GI bacteriomes of human and animals as well as environmental bacteriomes [10–12]. However, to date, there are only a few studies that have attempted to characterize the diversity and composition of the GI bacteriome of chickens and turkeys [13–16]. These studies only reported a relatively small number of sequences per sample (<10,000 reads) resulting in low coverage and a still incomplete picture of the diversity within the poultry GI bacteriome.

It has been recognized that the 454 pyrosequencing technology generates sequencing errors, sequence artifacts, and chimeric sequences [18, 19]. Also, different 454 sequence analysis pipelines use different algorithms for sequence alignment, phylogenetic distance computation, and OTU clustering, leading to different results [18–22], including over- or underestimation of diversity and species (OTUs) richness [23]. Moreover, little attention has been given to the repeatability of the technology across different pyrosequencing runs even though variability between runs can occur [24]. Thus, the primary objective of this study was to characterize the composition of the GI bacteriome in commercial poultry species, (chicken and turkey) using the 454 pyrosequencing technique. The secondary objective was to evaluate the repeatability of the technique and the effects of different data processing pipelines.

## 2. Materials and Methods

### 2.1. Sample Collection

All animal protocols were approved by the Ohio Agricultural Research and Development Center Animal Care and Use Committee.

Five broiler chickens were randomly chosen from each of three flocks at six weeks of age, and eight turkeys were chosen from one flock at 14 weeks of age. Both the chicken flocks and the turkey flock reared at the poultry Research Farm located at the Ohio Agricultural Research and Development Center (OARDC), Wooster, Ohio. The chickens and turkeys were fed standard corn-soybean-meal-based diets that met or exceeded the NRC requirements [25] for each species. Cecal contents were collected from each bird and pooled by species (the samples were pooled to reduce the number of samples while achieving high depth coverage per sample). The sequencing was done before Illumina sequencing was available. Individual ileal mucosa samples were collected from the region between Meckel's diverticulum and the ileocecal junction and pooled within species as described previously [26]. Each composite sample was mixed to represent each species and each GI region.

### 2.2. DNA Extraction and PCR

Community DNA was extracted from each of the four composite samples using the repeated bead-beating plus column purification method [27]. The V3 region (about 200 bp in length) of 16S rRNA gene in the metagenomic DNA was amplified with barcoded universal primer sets as listed in Table 1. Each forward primer consists of three parts: a 19 nt degenerated universal primer for bacterial 16S rRNA gene (357F), a 10 nt barcode, and the pyrosequencing adapter A. The reverse primer consisted of an 18 nt degenerated universal primer for bacterial 16S rRNA gene (519R) and a 19 nt pyrosequencing adapter B. The degenerate bases on primers were introduced to expand their inclusiveness.

For each PCR reaction, 400 ng of metagenomic DNA template was added to a 49 *μ*L master mix that contained 1x PCR buffer, 1.75 mM MgCl_2_, 670 ng/*μ*L bovine serum albumin, 200 *μ*M dNTP, 500 nM of each primer, and 0.625 U Platinum Taq DNA polymerase (Invitrogen Corporation, Carlsbad, CA). The PCR thermal program consisted of an initial denaturation at 95°C for 10 min; 20 or 25 cycles (20 cycles for cecal content samples and 25 cycles for ileal mucosa samples) of a 30 s denaturation step at 95°C, a 35 s annealing step at 55°C, and a 35 s elongation step at 72°C; and a final extension step at 72°C for 7 min, before a 4°C hold.

The quality of the PCR products was examined using agarose (1.2%) gel electrophoresis, and the expected PCR products of approximately 200 bp were gel purified using a Qiagen Gel Purification Kit (Qiagen, Valencia, CA, USA). The concentration of the purified products was quantified using a NanoDrop ND-1000 spectrophotometer (Thermo Scientific, Wilmington, DE) and confirmed using a Quant-it Kit (Invitrogen Corporation, Carlsbad, CA, USA).

### 2.3. Pyrosequencing and Data Analysis

Given the expected higher diversity in the cecal content samples compared with the ileal mucosa samples, the amplicons from the former and the latter were mixed in a 9 : 1 ratio for each species. The amplicons from the chickens and the turkeys were subsequently pooled in a 2 : 1 ratio. The pooled amplicon samples were divided and sequenced in two independent pyrosequencing runs on one-half of a picotiter plate each using a 454 Life Sciences Genome Sequencer FLX system (Roche, Basel, Switzerland) before the Genome Sequencer FLX Titanium became available. The V3 hypervariable region was sequenced because the FLX system produces a read length of less than 250 bp. The raw data were provided as sff files.

The quality of the 454 pyrosequencing data was evaluated using the raw sff data files following the standardized operating procedure (SOP) proposed by Schloss et al. [19]. Briefly, the flow file was generated from the sff file of each sample using the sffinfo program of the GS Analysis Software (Version 2.5, 454 Life Sciences Corporation, Branford, CT, USA). The pyrosequencing noise of each flow file was removed by the AmpliconNoise function implemented in Mothur [18, 19, 28]. The “denoised” sequences were trimmed off the primer 357F and 519R, which results in sequences of the V3 hypervariable region of 16S rRNA genes (minimum length: 100 bp, average length: 145 bp). The trimmed sequences were aligned using the Mothur aligner [28, 29] with the Silva_SSU_Ref_NR_108 dataset [30] as reference sequences and with a −4 score penalty for gap-pen and −3 score penalty for mismatch. Sequences that could not be aligned with the Silva reference dataset were removed. The common gaps in the sequence alignment were filtered out, and the sequences were preclustered to remove sequences that contain possible pyrosequencing errors [19, 31]. Possible chimeric sequences were identified and removed using UCHIME [32] implemented in the Mothur package [28].

A distance matrix of each dataset was computed using the ARB database environment with the Jukes-Cantor correction [30] applied. The Mothur and USEARCH were used to cluster sequences into OTUs at 0.03, 0.05, and 0.20 phylogenetic distances, generate rarefaction curves, and determine the nonparametric ACE and Chao1 estimates of maximum richness from each of the distance matrices. The maximum number of OTUs likely present in each of the samples was also estimated using the nonlinear models procedure (PROC NLIN) of SAS (V9.2, SAS Inst. Inc., Cary, NC), which fits the monomolecular function to the rarefaction output to determine the asymptote that serves as the upper bound of the curves as previously described [33]. Each distance matrix was computed three times, and the median values were chosen in calculating these indices to avoid under- or overestimation.

**Figure 1:**
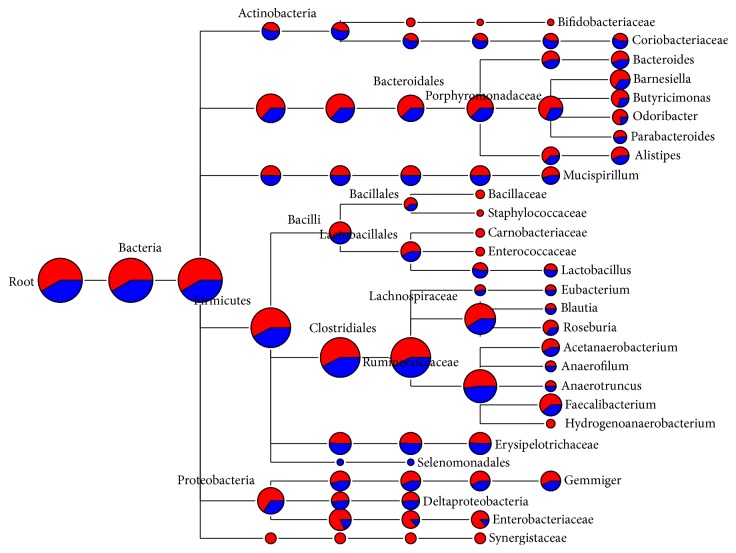
Bacterial diversity of chicken cecal content bacteriome. Only the genera represented by ≥5 OTUs each were shown, and the size of each node reflects the total number of OTUs. The relative proportion of taxa from the two pyrosequencing runs was shown by different colors: red, from the first run; blue, from the second run.

**Figure 2:**
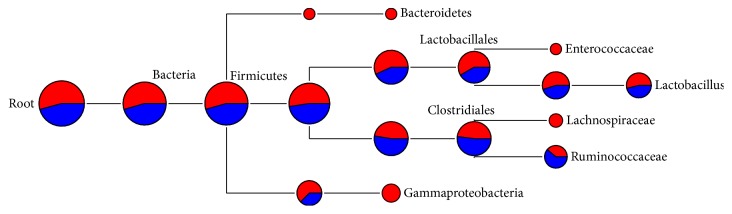
Bacterial diversity of chicken ileal mucosal bacteriome. Only the genera represented by ≥5 OTUs each were shown, and the size of each node reflects the total number of OTUs. The relative proportion of taxa from the two pyrosequencing runs was shown by different colors: red, from the first run; blue, from the second run.

**Figure 3:**
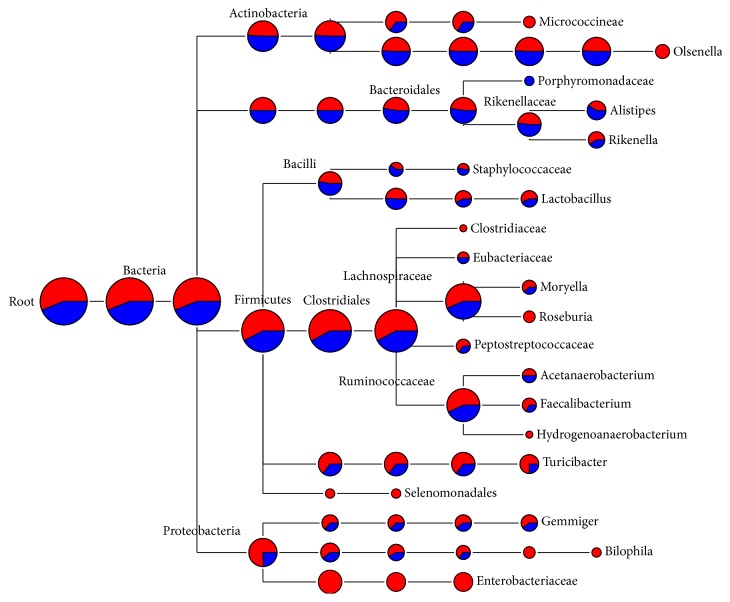
Bacterial diversity of turkey cecal content bacteriome. Only the genera represented by ≥5 OTUs each were shown, and the size of each node reflects the total number of OTUs. The relative proportion of taxa from the two pyrosequencing runs was shown by different colors: red, from the first run; blue, from the second run.

**Figure 4:**
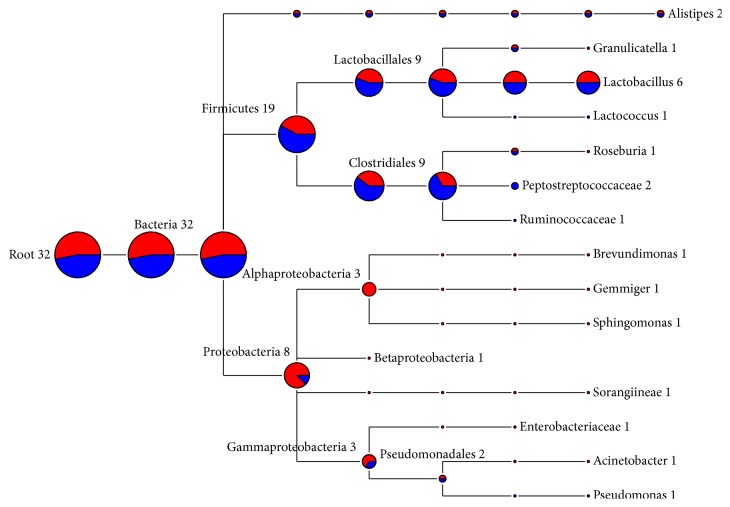
Bacterial diversity of turkey ileal mucosal bacteriome. Only the genera represented by ≥5 OTUs each were shown, and the size of each node reflects the total number of OTUs. The relative proportion of taxa from the two pyrosequencing runs was shown by different colors: red, from the first run; blue, from the second run.

**Figure 5:**
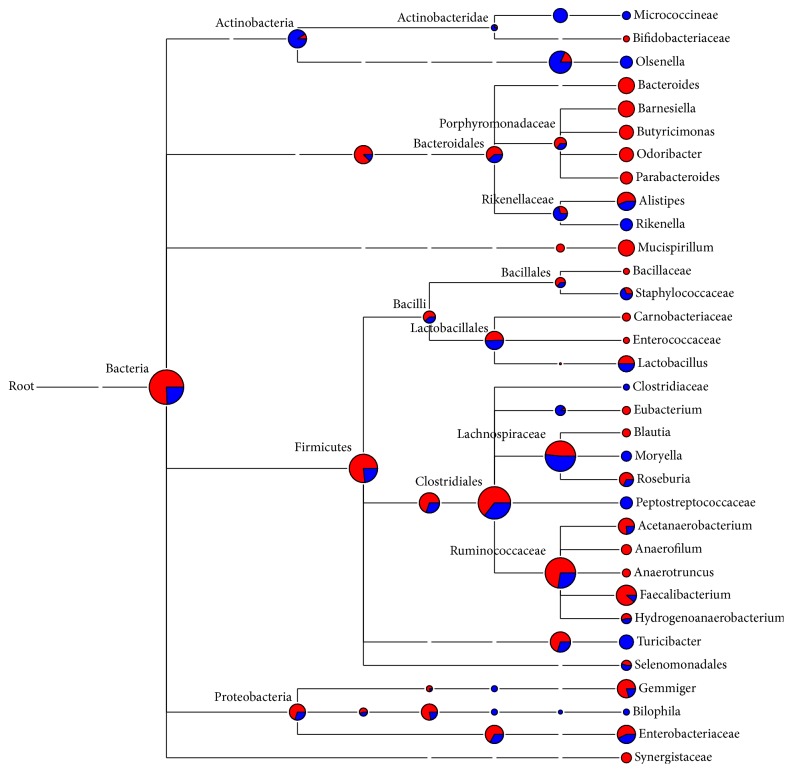
Comparison of the diversity of cecal content bacteriomes between broiler chickens and turkey. Only the genera represented by ≥5 OTUs each were shown, and the size of each node reflects the total number of OTUs from the four microbiomes. The proportion of each microbiome is distinguished by color: red, chicken; blue, turkey.

One representative sequence of each OTU defined at 0.03 genetic distances was obtained using the “get.oturep” command in the Mothur package V1.22. [28]. These OTU representative sequences were imported into ARB, and then a phylogenetic tree was constructed for each sample by inserting each sequence into the reference tree of the 286,858 Silva reference sequences (SSU_111_Ref_NR, http://www.arb-silva.de) as described previously [34]. The phylogenetic tree was then used for weighted UniFrac analysis as described in library comparison below. The sequences used in this study are maintained in an in-house ARB database dedicated to the GI bacteriome of chickens and turkey and is available from the corresponding author. The representative sequences of each sample were also archived in the MG-RAST server under the project of Poultry_MID_DB (4508915.3 to 4508920.3). The OTUs were classified using the RDP classifier [35] and the composition of each bacteriome was visualized as a taxonomic tree constructed using MEGAN [36]. Briefly, the taxonomic identification of 16S rRNA sequences was performed using the RDP naïve Bayesian classifier on the RDP server (https://rdp.cme.msu.edu/) [35] with the default setting. The taxonomy files were retrieved and imported into MEGAN using a minimum cutoff of 5 OTUs and a confidence score ≥ 50.0% [36]. The bacteriome composition of each gut location was shown as a hierarchical tree with the nodes showing the OTU counts (Figures 1–4). The bacteriome composition of cecal content was also compared between the chickens and the turkeys (Figure 5).

The GI bacteriomes of chickens and turkeys were compared using 4 different methods: weighted UniFrac distance, which measures the phylogenetic distance between sets of taxa as phylogenetic trees [37, 38]; the SONS function [39] in the Mothur package [28], which compares two bacteriomes by taking into consideration OTU richness, membership, and structure; RDP library comparison [40], which compares two libraries side by side based on the represented taxa and computes the likelihood that the frequency of membership in a given taxon is the same; and MEGAN phylogenetic tree [36], which allows comparison of bacteriomes based on detailed phylogenetic composition.

The numbers of raw sequence reads and quality-checked sequences were compared between the two independent pyrosequencing runs to assess the variation between the two runs. The quality-checked sequences were aligned using two different aligners in parallel to assess the effect of different aligners on OTUs clustering: the RDP Pyro aligner (http://pyro.cme.msu.edu/) against the Silva reference dataset provided on Mothur's webpage (https://www.mothur.org/) and the Mothur aligner. One distance matrix was computed for each alignment with the Jukes-Cantor correction applied, and OTUs were clustered using Mothur.

## 3. Results and Discussion

### 3.1. Overview of the 454 Pyrosequencing Results and the Variation between Runs

A total of 402,247 DNA sequence reads were obtained, of which 338,177 were successfully assigned to the corresponding samples based on the barcodes (Table 2). The sequencing data from the two pyrosequencing runs did not differ significantly regarding the number of raw sequences or sequences resulting from the denoising step. However, after the preclustering step, which was designed to reduce the effect of pyrosequencing errors [31], the second pyrosequencing run resulted in approximately 55% fewer sequence reads and 82% fewer denoised sequences. The coverage of diversity and composition of the GI bacteria also differed between the two pyrosequencing runs on the same sample set even though the numbers of raw reads were very similar. These results suggest that considerable variability in sequencing quality and numbers of usable sequences between different runs of the same pyrosequencing system corroborate a previous report [24]. Because the same amplicon libraries were used, the variability was produced during the sequencing process. Such run-to-run variations were also reported for the MiSeq technology [41].

The RDP Pyro aligner and Mothur aligner both align 16S rRNA sequences based on their secondary structure and can align a massive number of sequences relatively quickly [23, 29, 40, 42]. The RDP Pyro aligner does not align the hypervariable regions while the Mothur aligner does using the Silva alignment as reference sequences. Both aligners have been commonly used in analysis of pyrosequencing data. Thus, the two aligners were evaluated using the same dataset. The Mothur aligner resulted in approximately twice as many OTUs with the default alignment setting when penalties were given to gap open and mismatch. The RDP aligner yielded > 3-fold more OTUs than the Mothur aligner when the penalty options were applied. The RDP aligner has been reported to result in more OTUs from all but the V3 and V4 hypervariable regions of 16S rRNA genes when compared to the Silva aligner [19]. In this study, the V3 region was used, and more OTUs also resulted from the RDP Pyro aligner. In addition, even though the SOP of Mothur includes several quality checking procedures, the default setting may not be optimal for every dataset. As demonstrated in this study, the introduction of penalties for gap open and mismatch can significantly reduce the likelihood of overestimating diversity. It is recommended that in future studies more than one setting be used in each step to avoid inflation of diversity. Hence, the Mothur aligned sequences with penalties for gap open and mismatch applied were used in assessing the effect of different clustering algorithms on OTUs clustering in the present study.

Species richness of metagenomic datasets is typically expressed as numbers of OTUs clustered at a specific phylogenetic distance (commonly 0.03). In this study, Mothur and USEARCH, both of which are commonly used in OTU clustering [28, 43, 44], were compared using the same dataset of all four bacteriome samples. The USEARCH method generated twice as many OTUs as Mothur (Table 2). These results suggest that different clustering methods can produce different estimates of species richness, and thus comparisons of results from various studies, especially those that used different clustering and alignment methods, should be done with caution. Additionally, different phylogenetic distances might be needed when different clustering methods are used to produce comparable species richness. In this study, the Mothur was used to cluster OTUs from all the four datasets because it probably did not overestimate species richness.

### 3.2. Bacterial Diversity of Chicken Cecal Content Bacteriome

The cecum is the largest major reservoir of bacteria in poultry. The first and the second pyrosequencing runs produced 3,973 and 2,829 OTUs, respectively, from the chicken cecal samples (Table 2). The estimated asymptotes of OTUs reached 1.5-fold of the number of observed OTUs. Both pyrosequencing runs achieved a high level of coverage (Good's coverage > 95%) of the bacterial diversity in the chicken cecal bacteriome. The RDP classification of the OTUs from both pyrosequencing runs was combined and imported into MEGAN to generate a taxonomic tree of the major bacteria (Figure 1). In total, we identified nine bacterial phyla (Firmicutes, Proteobacteria, Bacteroides, Synergistetes, Fusobacteria, Actinobacteria, Deferribacteres, Tenericutes, and Lentisphaerae) and 84 known genera (data not shown). The Firmicutes was the most predominant phylum, accounting for 57.8% of the total bacterial sequences of the cecal content sample. Within this phylum, 30.9% of the OTUs could not be classified to any known taxa. The Bacteroidetes and Proteobacteria were far less predominant, accounting for 5.4% and 4.3% of the total bacteria sequences, respectively. No significant difference was observed at phylum or class level between the two pyrosequencing runs except for the class Gammaproteobacteria. However, a major difference was observed in unclassified Enterobacteriaceae. In the first pyrosequencing run,* Bifidobacteriaceae, *Bacillaceae, Staphylococcaceae, Carnobacteriaceae, Enterococcaceae, Hydrogenoanaerobacterium, and Synergistaceae were found to be represented by ≥5 OTUs each, while Selenomonadales was the only taxon that was represented by >5 OTUs. Consistent with a previous study [24], noticeable variation in diversity estimates can arise from different runs and quantitative interpretation of pyrosequencing data should be done with caution. Because the same amplicon libraries were sequenced in the two pyrosequencing runs and the sequence data were analyzed using the same sequence pipeline and parameters, the variations between the two pyrosequencing runs probably have arisen from the sequencing process.

A recent study established a global diversity framework of the poultry GI bacteriome by using a naïve analysis of all the 16S rRNA gene sequences generated from poultry GI (primarily cecal) bacteria that have been recovered worldwide using the Sanger sequencing technology [34]. When compared to this global cecal bacterial diversity database, there were 45 genera of bacteria in that database that were also found in the present study (Supplementary Table 1, in Supplementary Material available online at http://dx.doi.org/10.1155/2016/4320412). However, 29 genera were missing from the current pyrosequencing study (Supplementary Table 2). These include* Salmonella*,* Megamonas*, and* Paraprevotella*, each of which was represented by >10 sequences in that databases. On the other hand, the current pyrosequencing study identified 39 bacterial genera that were not represented in the global cecal bacterial diversity database, including* Butyricimonas, Odoribacter, Hydrogenoanaerobacterium, Moryella, Parasporobacterium*, and* Ruminococcus* (Supplementary Table 3). There were also many minor genera represented by less than five OTUs each. Overall, this study expanded the number of bacterial genera identified in the cecum of chickens by 80%. This is likely attributed to the increased sequencing depth we intentionally achieved. Most of these new genera found in the cecum might be present at low abundance. Future studies can further elucidate their importance to the host.

### 3.3. Bacterial Diversity of Chicken Ileal Mucosal Bacteriome

In this study, >5,000 sequences were obtained from each of the two pyrosequencing runs, resulting in 135 and 114 OTUs in the first and second runs, respectively (Table 2). Although the Good's coverage also reached >95%, the estimated asymptotes of OTUs were 1.9-fold greater than the observed numbers of OTUs. These results suggest that the diversity in the ileal mucosa has not been completely identified. It should be noted that approximately half of the original sequencing reads from the ileal mucosa appeared to be 18S rRNA genes of the host. The presence of these host sequences was probably due to the broad specificity of primers 357f and 519r, both of which can anneal to 18S rRNA genes [45]. Bacterial domain-specific primers can reduce or eliminate amplification of host 18S rRNA genes.

The OTUs from the chicken ileal mucosa were classified into seven bacterial phyla: Actinobacteria, Bacteroidetes, Cyanobacteria/Chloroplast, Firmicutes, Proteobacteria, Synergistetes, and Saccharibacteria. Firmicutes and Proteobacteria were the major phyla, accounting for 72.6% and 11.1% of the total sequences of the chicken ileal mucosa, respectively (Figure 2). For the predominant taxa, significant differences between the two pyrosequencing runs were not observed at taxonomic ranks from phylum to genus. However, some minor groups were only identified in one of the two pyrosequencing runs. For example, Bacteroidetes, Enterococcaceae, Lachnospiraceae, and Gammaproteobacteria were only identified in the first run, and each was represented by at least 5 OTUs, whereas* Prevotella*,* Salinicoccus*, and another 10 genera, each of which was represented by one or two OTUs, were only identified in the second run.* Lactobacillus* was the largest genus in the chicken ileal mucosa, accounting for 11% of the total sequences. In a previous study using 16S rRNA gene clone libraries,* Lactobacillus* was found to account for 75% of the ileal mucosal bacterial sequences among 7-day-old chicks [26]. The differences in bird age and methodologies used might explain the discrepancy in* Lactobacillus* predominance witnessed in the ileal mucosa. In a study in Australia, approximately 99% of the bacterial sequences from jejunal mucosa were classified to* Lactobacillus* [46]. Choices of methodology and target regions of the 16S rRNA gene may influence the observed relative abundance of lactobacilli in the gut of chickens. A greater predominance of* Lactobacillus* was expected for the jejunum than for the ileum.

### 3.4. Bacterial Diversity of Turkey Cecal Content Bacteriome

From the first and the second pyrosequencing runs, 1,891 and 1,481 OTUs were obtained, respectively (Table 2). Similar to the chicken cecal bacteriome, the estimated OTU asymptote of the turkey cecal bacteriome was approximately 1.5-fold greater than the number of OTUs observed, while the Good's coverage reached >95%. The OTUs were classified into eight bacterial phyla (Firmicutes, Bacteroides, Actinobacteria, Proteobacteria, Verrucomicrobia, Synergistetes, Elusimicrobia, and Lentisphaerae) and 85 known genera (data not shown). The phyla represented by ≥ 5 OTUs each are shown on the taxonomy tree (Figure 3). Firmicutes, Proteobacteria, and Actinobacteria were the most predominant phyla in the turkey cecal bacteriome, accounting for 66.3%, 7.4%, and 3.2% of the total bacterial sequences identified. Based on RDP Library comparison, a significant difference in the proportions of the phyla Firmicutes and Proteobacteria was observed between the two pyrosequencing runs. In total, 17 genera or groups were recovered only in the first sequencing run, including* Olsenella*, Clostridiaceae,* Roseburia*, Hydrogenoanaerobacterium, Selenomonadales,* Bilophila*, Enterobacteriaceae, and other groups, each of which was represented by ≤5 OTUs (Figure 3). On the other hand, 14 genera or groups were identified only in the second sequencing run, including Porphyromonadaceae and other minor genera. Only one OTU was classified as* Escherichia/Shigella,* a common genus of enteric bacteria, and this might be due to its low abundance in the turkey cecal bacteriome. Quantitative PCR analysis for this OTU may help confirm its abundance in the turkey cecal bacteriome.

The bacterial profile of the turkey cecal bacteriome was also compared to the global bacterial diversity framework of poultry [34]. Twenty-one genera, including* Megamonas*,* Prevotella*,* Paraprevotella*,* Subdoligranulum*,* Hallella*,* Phascolarctobacterium*, and minor genera representing less than ten sequences documented in the global dataset were not detected in the current pyrosequencing study. On the other hand, the current study uncovered 50 bacterial genera that were not represented in the global dataset. The major new genera include* Gemmiger*,* Olsenella*,* Moryella*,* Bilophila*,* Hydrogenoanaerobacterium*,* Akkermansia*,* Collinsella*,* Staphylococcus*,* Ruminococcus*,* Slackia*,* Sporacetigenium*, and some genera each represented by less than 5 OTUs. Future studies are needed to further understand the importance and contribution of these new genera to the overall intestinal health and nutrient utilization in turkeys. It should be noted, however, that some of the identified bacterial genera contain food-borne pathogens, such as* Bilophila*, which is implicated in several types of infection such as perforated and gangrenous appendicitis [47]. Thus, deep sequencing analysis not only facilitates a better understanding of the turkey cecal bacteriome but also provides an opportunity to identify potential risk factors that might have been overlooked. Future studies are needed to understand the factors that govern the populations of these pathogens.

### 3.5. Bacterial Diversity of Turkey Ileal Mucosal Bacteriome

This study is the first reported investigation of the turkey ileal mucosal bacteriome using pyrosequencing analysis. Most of the sequencing reads turned out to be host 18S rRNA gene sequences rather than bacterial 16S rRNA genes (Table 2). This is likely attributed to the low proportion of bacterial DNA in the DNA extract and also possibly the small number of PCR cycles (25 cycles). In total, only 30 bacterial 16S rRNA gene sequences were obtained from each of the two pyrosequencing runs, resulting in <20 OTUs (Table 2). The recovered OTUs was classified to the phyla Firmicutes, Proteobacteria, and Bacteroidetes, representing 59.3%, 25.0%, and 6.3% of the total bacterial sequences, respectively (Figure 4). Twelve genera of bacteria were found in the ileal mucosa of the turkeys. No significant differences between the two pyrosequencing runs were observed due to the small datasets, but seven genera were identified in the first pyrosequencing run, and only three genera were identified in the second sequencing run.

Except for* Lactobacillus*, all the identified genera were each represented by only one OTU.* Lactobacillus* and* Alistipes* were found in both pyrosequencing runs. The coverage of bacterial diversity in the turkey ileal mucosal bacteriome was far from complete, and thus the bacterial diversity of the turkey ileal mucosal bacteriome is not discussed any further in this paper. Again, future studies need to use primers that maximize amplification of bacterial 16S rRNA genes while reducing amplification of host DNA using bacteria domain-specific primers.

### 3.6. Comparisons between Chicken and Turkey Bacteriomes

The OTUs from the two pyrosequencing runs were combined, and the bacteriome profiles of the two species were then compared. Overall, the GI bacteriome of chickens appeared to be considerably different from that of turkeys when compared using UniFrac significance analysis (*p* < 0.01, data not shown). When compared on OTU richness, membership, and structure using the SONS analysis, the two GI bacteriomes were also distinct from each other, sharing only 18.6% Yue and Clayton index (*θ*_yc_ similarity) [17] at 0.03 distance level and 60.0% at 0.20 distance level (Table 3). As expected, greater community similarities were noted at higher phylogenetic distances.

The weighted UniFrac distances computed from the OTU representatives were used to access the structure of the poultry GI bacteriomes (Table 3). The weighted UniFrac distance was chosen over OTU-based approaches because the latter lacks the resolution to detect overlapping species between bacteriomes when datasets with low sequence coverage were used, while the former is a reliable index when comparing sequencing datasets with varied sample sizes [38]. Consistent with the analysis using SONS, the analysis using weighted UniFrac distances indicated that bacteriomes from the same GI region of the two species (chicken cecal content versus turkey cecal content) shared a greater similarity in phylogenetic structure than the bacteriomes from the different GI rejoins (ileal mucosa versus cecal content) of the same species. This is not surprising given the large differences in niche between cecal content and ileal mucosa.

Much smaller species richness was found in the ileal mucosal bacteriome of both chickens and turkeys (Table 2). About 95% of the bacteriome diversity was uncovered, but only a relatively small portion of the diversity in the ileal mucosal bacteriome of the turkeys was represented by the 16S rRNA gene sequences. Because the turkey ileal mucosal bacteriome is not adequately characterized, comparison of the ileal mucosal bacteriome was not included in this paper. However, the comparison of the ileal mucosal bacterial diversity between these two bird species and a comparative phylogenetic tree are available upon request.

The distribution and relative abundance of the major bacterial genera in the cecum differed between chickens and turkeys (Figure 5). Although the cecal bacteriomes of both species shared the major phyla (Actinobacteria, Bacteroidetes, Firmicutes, and Proteobacteria), significant differences were revealed in Firmicutes, Actinobacteria, and Bacteroidetes by RDP library comparison (data not shown). Fusobacteria and Deferribacteres were minor phyla that were only identified in the chicken cecal bacteriome, whereas Verrucomicrobia and Elusimicrobia were minor phyla only identified in the turkey cecal bacteriome. At the genus level,* Barnesiella* and* Odoribacter *were significantly more abundant in the chicken cecal bacteriome while the opposite was observed for* Olsenella *and* Rikenella *in the turkey cecal bacteriome. In total, 22 major genera (each represented by >5 OTUs) were only identified in chicken cecal bacteriome, including* Mucispirillum* and* Phascolarctobacterium *as the most predominant genera (in descending order), whereas 23 major genera were only identified in the turkey cecal bacteriome, including* Olsenella, Akkermansia, *and* Sporacetigenium *as the most predominant genera. It is also of interest to note the different occurrence of* Actinobacteria *genera between the two bird species: Micrococcineae and Coriobacteriaceae were the predominant taxa in the turkey cecal bacteriome, while Bifidobacteriaceae of Actinobacteria was predominant in the chicken cecal bacteriome. Such a disparity needs to be confirmed, and their implications in host nutrition and health warrant further investigation.

As discussed earlier [1, 34, 48], differences in host (genetics, breeds, anatomical features of the gut, physiology, etc.), litter management, and diets might be attributable to the distinct GI bacteriomes in the chicken and turkey. For example, turkeys have a larger intestinal diameter, more viscous digesta, and a slower digesta passage rate (i.e., longer retention time) than chickens [49]. These factors may lead to lower partial O_2_ pressure and redox potential in the gut of turkeys compared to that of chickens. Diet, however, is one the major factors affecting GI bacteriome in poultry [1]. The chickens in US commercial farms are typically fed corn-soybean-based diets that meet the NRC requirement, so were the chickens used in the present study. Thus, we compared the results from the present study with those of published studies where chickens were also fed corn-soybean-based diet. In the study of Lumpkin et al. [50], the bacteriome of three genetic lines of chickens were examined using DGGE and T-RFLP, and Lactobacillaceae, Clostridiaceae, Enterococcaceae, and Bacteroidaceae were the major bacterial families detected in the cecum of chickens at both 4 and 35 days of age. Staphylococcaceae was also detected in multipurpose broilers. These five families were well represented in the chicken cecal bacteriome of the present study (Figure 1). Owing to the limited resolution, only five major bacterial families were detected in the study by Lumpkins et al. [50]. Our pyrosequencing analysis enabled detailed characterization of the bacteriome at OTU level. In one study on broiler chicken was challenged with* Clostridium perfringens*; the bacteriome was profiled using low depth coverage (6-7K sequences per sample) pyrosequencing of the V3–V5 region [51]. In the unchallenged control group fed corn-soybean-based diet, only 2-3 major (>2%) bacterial families or orders were found. At day 13, the control group was predominated by* Clostridiaceae* (2.41%), Lactobacillaceae (80.00%), and Enterobacteriaceae (11.99%), or by* Clostridiales* (85.17%) and Lactobacillaceae (7.13%). We identify all the above major bacterial families, suggesting that the bacteriome revealed in the present study is representative of the chickens reared in commercial settings.

The diets used in studies on turkey GI bacteriome were not as well documented as those used in studies on the chicken GI bacteriome. We did a literature search of the PubMed database with keyword “turkey,” “corn-soybean-based,” and “bacteriome” (or “microbiome” and “microbiota”) but found no publication. However, a few studies examined the turkey GI microbiota [52, 53]. Based on Illumina sequencing of the V3 region of 16S rRNA gene [52], the turkey cecal microbiome was dominated by Clostridia (~90% of total bacterial sequences), while as poults grow, Bacilli, Actinobacteria, and Bacteroidia grew in predominance (5–20%). The turkeys used in the present study were fed a corn-soybean-based diet and reared under commercial turkey management conditions. The above taxa, along with other taxa, were also detected in the present study, suggesting that the turkey GI bacteriome revealed in the present study approximates the actual GI bacteriome of turkeys reared in commercial settings. Therefore, this metagenomic study added new information to the poultry intestinal bacteriome and may facilitate future studies. It remains to be determined to what extent the distinct GI bacteriome of these two bird species contributes differently to host health and nutrient utilization.

## 4. Conclusion

A large number of genera and OTUs were found in the cecum and ileal mucosa of broiler chickens and turkeys, expanding our knowledge on the GI bacteriome of these two bird species, especially when they are reared under the dietary and managerial conditions common in North America. Some of the bacterial groups unique to each bird species might be important to host health and performance. A comprehensive knowledge of the GI bacteriome of chickens and turkeys and the differences between these bird species can be useful in modulating GI bacteriome to improve host health and growth performance.

## Supplementary Material

Suppl. Table 1. Bacterial genera that were represented both in the global diversity database of chicken cecal bacteria and in this study. A detailed list of the 45 genera identified in both global diversity database and in this study. The global diversity database was established by using a naïve analysis of all the 16S rRNA gene sequences originated from poultry GI (primarily cecal) bacteria that have been recovered worldwide using the Sanger sequencing technology. The taxonomic ranks above genus, the genus, and the numbers of representative sequences were listed for a side-by-side comparison between the global diversity database and this study.
Suppl. Table 2. Bacterial genera not detected in this study but represented in the global diversity database of chicken cecal bacteria. A detailed list of the 29 genera of bacteria that were not recovered in this study, but present in the global diversity database of chicken cecal bacteria. The taxonomic ranks above genus, the genus, and the numbers of representative sequences were listed.
Suppl. Table 3. Bacterial genera detected in this study but not represented in the global diversity database of chicken cecal bacteria. A detailed list of the 39 genera of bacteria that were recovered in this study, but not present in the global diversity database of chicken cecal bacteria. The taxonomic ranks above genus, the genus, and the numbers of representative sequences were listed.

## Figures and Tables

**Table 1:** Barcoded degenerate primers used to produce the V3 amplicon libraries.

*Forward*	B-A-D16-357F: GCCTCCCTCGCGCCATCAGACGCTCGACACWYCTACGGRDGGCWGCAG
	C-A-D16-357F: GCCTCCCTCGCGCCATCAGAGACGCACTCCWYCTACGGRDGGCWGCAG
	D-A-D16-357F: GCCTCCCTCGCGCCATCAGAGCACTGTAGCWYCTACGGRDGGCWGCAG
	E-A-D16-357F: GCCTCCCTCGCGCCATCAGATCAGACACGCWYCTACGGRDGGCWGCAG
*Reverse*	B-D4-519R: GCCTTGCCAGCCCGCTCAGGTNTTACCGCGGCTGCTG

The unique barcode is underlined.

**Table 2:** Summary of the 454 pyrosequencing data.

Sample	# of raw seqs assigned	# of sequences after screening	# of preclustered seqs after screening	Observed OTUs		Maximum # of OTUs			Good's coverage^*∗∗*^
				Mothur	USEARCH	Rarefaction asymptote^*∗*^	Chao1	ACE	
CD-1	98021	91456	21080	3973	8899	6001	10657	19318	95.7%
CD-2	98238	91227	12437	2829	5162	4278	7806	14361	96.9%
CM-1	5457	2714	598	135	324	259	370	1121	95.0%
CM-2	5273	2506	330	114	199	205	287	580	95.5%
TD-1	56959	53527	10304	1891	4252	2779	5114	9286	96.5%
TD-2	57442	49836	6685	1484	2706	2188	3971	6948	97.0%
TM-1	8104	30	23	17	19	63	63	246	43.3%
TM-2	8683	33	19	15	16	19	18	21	54.5%

CD, chicken cecal digesta; CM, chicken ileal mucosa; TD, turkey cecal digesta; TM, turkey ileal mucosa.

^**∗**^Estimated by Number  of  phylotypes = *α*(1 − *β* × *e*^[−*κ* × *n*]^).

^*∗∗*^Estimated by Coverage  of  diversity = (*n* − *N*)/*n* × 100%.

**Table 3:** Comparisons of intestinal bacterial diversity between chickens and turkeys.

Source	Distance level	# of OTUs shared	*θ*_yc_^b^ (lci, hci)	UniFrac distance
*CD vs CM*	0.03	74	0.030 (0.023, 0.038)	0.627
	0.05	76	0.052 (0.034, 0.068)	
	0.20	31	0.280 (0.211, 0.350)	
*CD vs TD*	0.03	743	0.186 (0.170, 0.202)	0.364
	0.05	758	0.332 (0.303, 0.358)	
	0.20	69	0.600 (0.564, 0.630)	

b: *θ*_yc_ = ∑_*i*=1_^*S*_*T*_^*a*_*i*_*b*_*i*_/(∑_*i*=1_^*S*_*T*_^(*a*_*i*_ − *b*_*i*_)^2^ + ∑_*i*=1_^*S*_*T*_^*a*_*i*_*b*_*i*_) [17]

where,

*S*_*T*_ is the total number of OTUs in communities A and B;

*a*_*i*_ is the relative abundance of OTU *i* in community A;

*b*_*i*_ is the relative abundance of OTU *i* in community B.
