# Supplementary material for: The Bacteriomes of Ileal Mucosa and Cecal Content of Broiler Chickens and Turkeys as Revealed by Metagenomic Analysis
Source: Int J Microbiol. 2016 Dec 28;2016:4320412. doi: 10.1155/2016/4320412 (PMC5225337; doi:10.1155/2016/4320412)
Supplement: Supplementary file 1 — Suppl. Table 1. Bacterial genera that were represented both in the global diversity database of chicken cecal bacteria and in this study. A detailed list of the 45 genera identified in both global diversity database and in this study. The global diversity database was established by using a naïve analysis of all the 16S rRNA gene sequences originated from poultry GI (primarily cecal) bacteria that have been recovered worldwide using the Sanger sequencing technology. The taxonomic ranks above genus, the genus, and the numbers of representative sequences were listed for a side-by-side comparison between the global diversity database and this study. Suppl. Table 2. Bacterial genera not detected in this study but represented in the global diversity database of chicken cecal bacteria. A detailed list of the 29 genera of bacteria that were not recovered in this study, but present in the global diversity database of chicken cecal bacteria. The taxonomic ranks above genus, the genus, and the numbers of representative sequences were listed. Suppl. Table 3. Bacterial genera detected in this study but not represented in the global diversity database of chicken cecal bacteria. A detailed list of the 39 genera of bacteria that were recovered in this study, but not present in the global diversity database of chicken cecal bacteria. The taxonomic ranks above genus, the genus, and the numbers of representative sequences were listed. [file 4320412.f1.pdf]

Suppl. Table 1. Bacterial genera that were represented both in the global diversity database of chicken cecal bacteria and in this study

**Lineage**	**Genus**	**# of Seqs in global database**	**# of Seqs in this study**
Firmicutes;Clostridia;Clostridiales;Clostridiaceae 1	*Clostridium sensu stricto*	1	1
Actinobacteria;Actinobacteria;Coriobacteridae;Coriobacteriales; Coriobacterineae;Coriobacteriaceae	*Enterorhabdus*	1	1
Tenericutes;Mollicutes;Anaeroplasmatales;Anaeroplasmataceae	*Anaeroplasma*	1	2
Proteobacteria;Gammaproteobacteria;Enterobacteriales;Enterobacteriaceae	*Proteus*	1	2
Firmicutes;Clostridia;Clostridiales;Eubacteriaceae	*Eubacterium*	1	10
Firmicutes;Clostridia;Clostridiales;Ruminococcaceae	*Acetanaerobacterium*	1	12
Deferribacteres;Deferribacteres;Deferribacterales;Deferribacteraceae	*Mucispirillum*	1	46
Proteobacteria;Betaproteobacteria;Burkholderiales;Sutterellaceae	*Parasutterella*	2	2
Firmicutes;Clostridia;Clostridiales;Ruminococcaceae	*Sporobacter*	2	2
Proteobacteria;Epsilonproteobacteria;Campylobacterales;Helicobacteraceae	*Helicobacter*	2	5
Firmicutes;Clostridia;Clostridiales;Ruminococcaceae	*Anaerofilum*	2	12
Firmicutes;Clostridia;Clostridiales;Peptostreptococcaceae	*Clostridium XI*	3	1
Actinobacteria;Actinobacteria;Actinobacteridae;Bifidobacteriales; Bifidobacteriaceae	*Bifidobacterium*	3	2
Proteobacteria;Deltaproteobacteria;Desulfovibrionales;Desulfovibrionaceae	*Desulfovibrio*	3	2
Proteobacteria;Deltaproteobacteria;Desulfovibrionales;Desulfovibrionaceae	*Bilophila*	3	5
Bacteroidetes;Bacteroidia;Bacteroidales;Porphyromonadaceae	*Parabacteroides*	3	17
Firmicutes;Clostridia;Clostridiales;Lachnospiraceae	*Roseburia*	3	23
Firmicutes;Erysipelotrichia;Erysipelotrichales;Erysipelotrichaceae	*Coprobacillus*	4	2
Firmicutes;Erysipelotrichia;Erysipelotrichales;Erysipelotrichaceae	*Clostridium XVIII*	4	34
Firmicutes;Bacilli;Lactobacillales;Enterococcaceae	*Enterococcus*	5	6
Bacteroidetes;Bacteroidia;Bacteroidales;Porphyromonadaceae	*Barnesiella*	5	62
Firmicutes;Clostridia;Clostridiales;Lachnospiraceae	*Coprococcus*	6	6
Firmicutes;Clostridia;Clostridiales;Lachnospiraceae	*Dorea*	8	1
Firmicutes;Clostridia;Clostridiales;Ruminococcaceae	*Flavonifractor*	8	110
Bacteroidetes;Bacteroidia;Bacteroidales;Rikenellaceae	*Rikenella*	9	1
Firmicutes;Erysipelotrichia;Erysipelotrichales;Erysipelotrichaceae	*Erysipelotrichaceae _incertae_sedis*	9	24
Actinobacteria;Actinobacteria;Coriobacteridae;Coriobacteriales; Coriobacterineae;Coriobacteriaceae	*Collinsella*	10	5
Firmicutes;Clostridia;Clostridiales;Ruminococcaceae	*Anaerotruncus*	10	13
Firmicutes;Clostridia;Clostridiales;Lachnospiraceae	*Clostridium XlVb*	10	38

Firmicutes;Clostridia;Clostridiales;Ruminococcaceae	*Pseudoflavonifractor*	10	68
Firmicutes;Clostridia;Clostridiales;Ruminococcaceae	*Acetivibrio*	12	4
Firmicutes;Negativicutes;Selenomonadales;Acidaminococcaceae	*Phascolarctobacterium*	15	7
Proteobacteria;Alphaproteobacteria;Rhizobiales;Hyphomicrobiaceae	*Gemmiger*	15	37
Firmicutes;Clostridia;Clostridiales;Ruminococcaceae	*Butyricicoccus*	15	68
Firmicutes;Clostridia;Clostridiales;Ruminococcaceae	*Oscillibacter*	16	69
Bacteroidetes;Bacteroidia;Bacteroidales;Rikenellaceae	*Alistipes*	18	37
Firmicutes;Clostridia;Clostridiales;Ruminococcaceae	*Subdoligranulum*	19	6
Firmicutes;Clostridia;Clostridiales;Lachnospiraceae	*Blautia*	28	10
Firmicutes;Bacilli;Lactobacillales;Lactobacillaceae	*Lactobacillus*	32	21
Proteobacteria;Gammaproteobacteria;Enterobacteriales;Enterobacteriaceae	*Escherichia/Shigella*	33	4
Firmicutes;Clostridia;Clostridiales;Lachnospiraceae	*Clostridium XlVa*	39	3
Bacteroidetes;Bacteroidia;Bacteroidales;Bacteroidaceae	*Bacteroides*	45	41
Firmicutes;Clostridia;Clostridiales;Ruminococcaceae	*Clostridium IV*	49	28
Firmicutes;Clostridia;Clostridiales;Ruminococcaceae	*Faecalibacterium*	54	114
Firmicutes;Clostridia;Clostridiales;Lachnospiraceae	*Lachnospiracea _incertae_sedis*	84	9

Suppl. Table 2. Bacterial genera not detected in this study but represented in the global diversity database of chicken cecal bacteria

**Lineage**	**Genus**	**# of Sequences**
Proteobacteria;Gammaproteobacteria;Enterobacteriales;Enterobacteriaceae	*Salmonella*	41
Firmicutes;Negativicutes;Selenomonadales;Veillonellaceae	*Megamonas*	22
Bacteroidetes;Bacteroidia;Bacteroidales;Prevotellaceae;	*Paraprevotella*	16
Actinobacteria;Actinobacteria;Coriobacteridae;Coriobacteriales;Coriobacterineae; Coriobacteriaceae	*Olsenella*	8
Proteobacteria;Gammaproteobacteria;Enterobacteriales;Enterobacteriaceae	*Klebsiella*	6
Firmicutes;Clostridia;Clostridiales;Lachnospiraceae	*Lactonifactor*	6
Firmicutes;Negativicutes;Selenomonadales;Veillonellaceae	*Megasphaera*	5
Proteobacteria;Gammaproteobacteria;Pseudomonadales;Pseudomonadaceae	*Pseudomonas*	5
Firmicutes;Clostridia;Clostridiales;Lachnospiraceae	*Robinsoniella*	5
Firmicutes;Clostridia;Clostridiales;Lachnospiraceae	*Anaerostipes*	4
Verrucomicrobia;Verrucomicrobiae;Verrucomicrobiales;Verrucomicrobiaceae	*Akkermansia*	3
Proteobacteria;Gammaproteobacteria;Enterobacteriales;Enterobacteriaceae	*Enterobacter*	3
Proteobacteria;Gammaproteobacteria;Aeromonadales;Succinivibrionaceae	*Succinatimonas*	3
Firmicutes;Clostridia;Clostridiales;Clostridiales_Incertae Sedis XIII	*Anaerovorax*	2
Actinobacteria;Actinobacteria;Coriobacteridae;Coriobacteriales;Coriobacterineae; Coriobacteriaceae	*Coriobacterium*	2
Bacteroidetes;Bacteroidia;Bacteroidales;Prevotellaceae	*Hallella*	2
Firmicutes;Clostridia;Clostridiales;Lachnospiraceae	*Hespellia*	2
Proteobacteria;Betaproteobacteria;Burkholderiales;Oxalobacteraceae	*Massilia*	2
Proteobacteria;Gammaproteobacteria;Xanthomonadales;Xanthomonadaceae	*Stenotrophomonas*	2
Proteobacteria;Betaproteobacteria;Burkholderiales;Sutterellaceae	*Sutterella*	2
Proteobacteria;Deltaproteobacteria;Bdellovibrionales;Bdellovibrionaceae	*Vampirovibrio*	2
Firmicutes;Negativicutes;Selenomonadales;Acidaminococcaceae	*Acidaminococcus*	1
Proteobacteria;Gammaproteobacteria;Aeromonadales;Aeromonadaceae	*Aeromonas*	1
Fusobacteria;Fusobacteria;Fusobacteriales;Fusobacteriaceae	*Clostridium XIX*	1
Proteobacteria;Gammaproteobacteria;Pasteurellales;Pasteurellaceae	*Gallibacterium*	1
Firmicutes;Clostridia;Clostridiales;Peptococcaceae 1	*Peptococcus*	1
Spirochaetes;Spirochaetes;Spirochaetales;Spirochaetaceae	*Spirochaeta*	1
Firmicutes;Clostridia;Clostridiales;Lachnospiraceae	*Syntrophococcus*	1
Proteobacteria;Gammaproteobacteria;Enterobacteriales;Enterobacteriaceae	*Trabulsiella*	1

Suppl. Table 3. Bacterial genera detected in this study but not represented in the global diversity database of chicken cecal bacteria

Lineage	Genus	# of Sequence
Bacteroidetes;Bacteroidia;Bacteroidales;Porphyromonadaceae	*Butyricimonas*	38
Bacteroidetes;Bacteroidia;Bacteroidales;Porphyromonadaceae	*Odoribacter*	26
Firmicutes;Clostridia;Clostridiales;Ruminococcaceae	*Hydrogenoanaerobacterium*	10
Firmicutes;Clostridia;Clostridiales;Lachnospiraceae	*Moryella*	7
Firmicutes;Clostridia;Clostridiales;Lachnospiraceae	*Parasporobacterium*	6
Firmicutes;Clostridia;Clostridiales;Ruminococcaceae	*Ruminococcus*	5
Firmicutes;Clostridia;Clostridiales;Ruminococcaceae	*Saccharofermentans*	5
Actinobacteria;Actinobacteria;Actinobacteridae;Bifidobacteriales; Bifidobacteriaceae	*Aeriscardovia*	4
Firmicutes;Clostridia;Clostridiales;Ruminococcaceae	*Clostridium III*	4
Firmicutes;Clostridia;Clostridiales;Ruminococcaceae	*Ethanoligenens*	4
Firmicutes;Clostridia;Clostridiales;Lachnospiraceae	*Marvinbryantia*	3
Firmicutes;Bacilli;Bacillales;Staphylococcaceae	*Salinicoccus*	3
Firmicutes;Bacilli;Lactobacillales;Streptococcaceae	*Streptococcus*	3
Firmicutes;Clostridia;Clostridiales;Incertae Sedis XI	*Anaerosphaera*	2
Actinobacteria;Actinobacteria;Coriobacteridae;Coriobacteriales; Coriobacterineae;Coriobacteriaceae	*Eggerthella*	2
Fusobacteria;Fusobacteria;Fusobacteriales;Fusobacteriaceae	*Fusobacterium*	2
Firmicutes;Clostridia;Clostridiales;Lachnospiraceae;	*Lachnospira*	2
Actinobacteria;Actinobacteria;Coriobacteridae;Coriobacteriales; Coriobacterineae;Coriobacteriaceae	*Slackia*	2
Firmicutes;Clostridia;Clostridiales;Lachnospiraceae	*Sporobacterium*	2
Firmicutes;Bacilli;Bacillales;Staphylococcaceae	*Staphylococcus*	2
Lentisphaerae;Lentisphaeria;Victivallales;Victivallaceae	*Victivallis*	2
Firmicutes;Erysipelotrichia;Erysipelotrichales;Erysipelotrichaceae	*Allobaculum*	1
Firmicutes;Bacilli;Lactobacillales;Carnobacteriaceae	*Atopostipes*	1
Firmicutes;Clostridia;Clostridiales;Lachnospiraceae	*Butyrivibrio*	1
Synergistetes;Synergistia;Synergistales;Synergistaceae	*Dethiosulfovibrio*	1
Actinobacteria;Actinobacteria;Actinobacteridae;Actinomycetales; Corynebacterineae;Dietziaceae	*Dietzia*	1
Firmicutes;Bacilli;Lactobacillales;Carnobacteriaceae	*Dolosigranulum*	1
Firmicutes;Clostridia;Clostridiales;Ruminococcaceae	*Fastidiosipila*	1
Firmicutes;Bacilli;Bacillales;Paenibacillaceae 1	*Fontibacillus*	1
Actinobacteria;Actinobacteria;Coriobacteridae;Coriobacteriales; Coriobacterineae;Coriobacteriaceae	*Gordonibacter*	1
Firmicutes;Erysipelotrichia;Erysipelotrichales;Erysipelotrichaceae	*Holdemania*	1
Firmicutes;Clostridia;Clostridiales;Gracilibacteraceae	*Lutispora*	1
Firmicutes;Bacilli;Bacillales;Staphylococcaceae	*Nosocomiicoccus*	1
Firmicutes;Clostridia;Clostridiales;Lachnospiraceae	*Oribacterium*	1
Firmicutes;Clostridia;Clostridiales;Syntrophomonadaceae	*Pelospora*	1
Firmicutes;Clostridia;Clostridiales;Eubacteriaceae	*Pseudoramibacter*	1
Firmicutes;Clostridia;Clostridiales;Clostridiaceae 1	*Sarcina*	1
Bacteroidetes;Bacteroidia;Bacteroidales;Porphyromonadaceae	*Tannerella*	1
Firmicutes;Clostridia;Clostridiales;Clostridiales_Incertae Sedis XI	*Tepidimicrobium*	1
